# Identification of key biomarkers for predicting CAD progression in inflammatory bowel disease via machine‐learning and bioinformatics strategies

**DOI:** 10.1111/jcmm.18175

**Published:** 2024-03-07

**Authors:** Xiaoqi Tang, Yufei Zhou, Zhuolin Chen, Chunjiang Liu, Zhifeng Wu, Yue Zhou, Fan Zhang, Xuanyuan Lu, Liming Tang

**Affiliations:** ^1^ School of Medicine Shaoxing University Zhejiang China; ^2^ Department of Cardiology Shanghai Institute of Cardiovascular Diseases, Zhongshan Hospital and Institutes of Biomedical Sciences, Fudan University Shanghai China; ^3^ Department of Orthopedics Shaoxing People's Hospital (Zhejiang University School of Medicine) Shaoxing China; ^4^ Department of General Surgery, Division of Vascular Surgery Shaoxing People's Hospital Shaoxing China

**Keywords:** acute myocardial infarction, atherosclerosis progression, bioinformatics, coronary artery disease, inflammatory bowel disease, inflammation and immunity, machine‐learning

## Abstract

The study aimed to identify the biomarkers for predicting coronary atherosclerotic lesions progression in patients with inflammatory bowel disease (IBD). Related transcriptome datasets were seized from Gene Expression Omnibus database. IBD‐related modules were identified via Weighted Gene Co‐expression Network Analysis. The ‘Limma’ was applied to screen differentially expressed genes between stable coronary artery disease (CAD) and acute myocardial infarction (AMI). Subsequently, we employed protein‐protein interaction (PPI) network and three machine‐learning strategies to further screen for candidate hub genes. Application of the receiver operating characteristics curve to quantitatively evaluate candidates to determine key diagnostic biomarkers, followed by a nomogram construction. Ultimately, we performed immune landscape analysis, single‐gene GSEA and prediction of target‐drugs. 3227 IBD‐related module genes and 570 DEGs accounting for AMI were recognized. Intersection yielded 85 shared genes and mostly enriched in immune and inflammatory pathways. After filtering through PPI network and multi‐machine learning algorithms, five candidate genes generated. Upon validation, CTSD, CEBPD, CYP27A1 were identified as key diagnostic biomarkers with a superior sensitivity and specificity (AUC > 0.8). Furthermore, all three genes were negatively correlated with CD4^+^ T cells and positively correlated with neutrophils. Single‐gene GSEA highlighted the importance of pathogen invasion, metabolism, immune and inflammation responses during the pathogenesis of AMI. Ten target‐drugs were predicted. The discovery of three peripheral blood biomarkers capable of predicting the risk of CAD proceeding into AMI in IBD patients. These identified biomarkers were negatively correlated with CD4^+^ T cells and positively correlated with neutrophils, indicating a latent therapeutic target.

## 1 INTRODUCTION

Inflammatory bowel disease (IBD), consisting of Crohn's disease (CD) and ulcerative colitis (UC), is an autoimmune disease which is characterized by compromised structure and function of gastrointestinal tract due to non‐specific chronic inflammation.^1^ During the past decades, the prevalence of IBDs have increased rapidly among different ethnics and regions, resulting in a huge challenge to healthcare worldwide.^2^ Emerging epidemiologic evidence indicated that immune‐mediated inflammatory disorders were involved in the occurrence and development of atherosclerotic cardiovascular disease, including rheumatoid arthritis, ankylosing spondylitis, IBD and systemic lupus erythematosus.^3^, ^4^, ^5^ In a meta‐analysis of 10 cohort studies, both CD (RR = 1.243; 95% CI 1.042–1.482) and UC (RR = 1.206; 95% CI 1.170–1.242) were significantly associated with an increased risk of ischemic heart disease compared to matched non‐IBD controls.^6^ Zanoli et al. showed that systemic chronic inflammation can stimulate endothelial dysfunction and artery stiffening in IBD individuals.^7^ Thus, it is required to enhance the attention on the cardiovascular diseases in patients with IBDs.

Coronary artery disease (CAD), is actually one of the major cardiovascular diseases affecting the global population, which has been reported to be the leading cause of mortality in both developed and developing countries.^8^ Traditional risk factors of CAD are previously characterized with dyslipidemia, high blood pressure, diabetes, smoking and family history of cardiovascular disorder.^9^, ^10^, ^11^, ^12^, ^13^ Overweight may also be a risk factor.^14^ This trouble is a lipoprotein‐driven disease with a waxy material known as atherosclerotic plaque builds up inside the heart's arteries.^15^ With decades of indolent progression, the plaque becomes enlarged and blocks the coronary blood vessels, and may predispose to an acute coronary syndrome caused by the plaque rupturing suddenly and superimposed thrombosis.^16^ If medical assistance failed to perform promptly, the affected myocardium encounter irreversible necrosis pathologically induced by prolonged ischemia and hypoxia.^17^ According to Centers for Disease Control and Prevention (CDC) (https://wonder.cdc.gov/), over 100 thousand people die from myocardial infarction every year in U.S. Despite advanced insights and various treatment options on the acute myocardial infarction (AMI) and other cardiovascular diseases, there are still challenges to differentiate AMI and stable CAD diagnose, particularly for silent MI without obviously typical symptoms.^18^ Therefore, it is necessary to explore peripheral blood diagnostic biomarkers in clinical practice for predicting and assessing the risk of CAD progression to AMI in advance.

CAD and IBD were usually regarded two distinct entities before. However, considerable evidence has accumulated to show that two diseases share common pathogenesis regarding to genetics, immunology, and contributing factors.^19^ Mutations in the nucleotide binding oligomerization domain containing 2 (NOD2) was identified the first susceptibility gene for increasing the risk of Crohn's disease.^20^ It mediates the host's immune response by sensing the intracellular bacterial muramyl dipeptide, leading to nucleus accumulation of pro‐inflammatory transcriptional regulator, NF‐κB.^21^ It is reported that NOD2 deficient mice predispose to atherosclerosis and heightened activation of TLR4, MAPKs and NF‐κB signal pathways compared to wild‐type mice.^22^, ^23^ These findings indicated that NOD2 polymorphisms exert a significant impact on the pathogenesis of both IBD and CAD. Like NOD2 gene, polymorphisms of the NLRP3, CDKN2B, stromelysin‐1 and even in the regulatory regions of inflammatory cytokines (e.g., IL‐6 rs1800795 and C −511T in the IL‐1*β* promoter region) were also associated with the two diseases.^19^, ^24^, ^25^, ^26^ Both IBD and CAD were involved in the innate immune pathways mediated by toll like receptors,^27^ as well as adaptive immunity maintained by elevated inflammatory T cell infiltration.^28^, ^29^ Additionally, smoking, diabetes, dysbiosis of gut microbiota and systematic inflammation were considered shared risk factors for atherosclerotic cardiovascular disease and IBD.^30^

Most previous studies only focused on the comparison of CAD or AMI versus healthy populations, but investigation for biomarkers that play a crucial role in the progression of CAD proceeding to AMI is limited, especially in IBD patients. Herein, by adopting multiple machine‐learning algorithms and bioinformatics strategy, the paper aims to explore potential biomarkers for predicting the CAD progression in patients with IBD and assess immune alterations, providing reference for future prevention and treatment.

## 2 MATERIALS AND METHODS

### 2.1 Study design and microarray data collection

**FIGURE 1:**
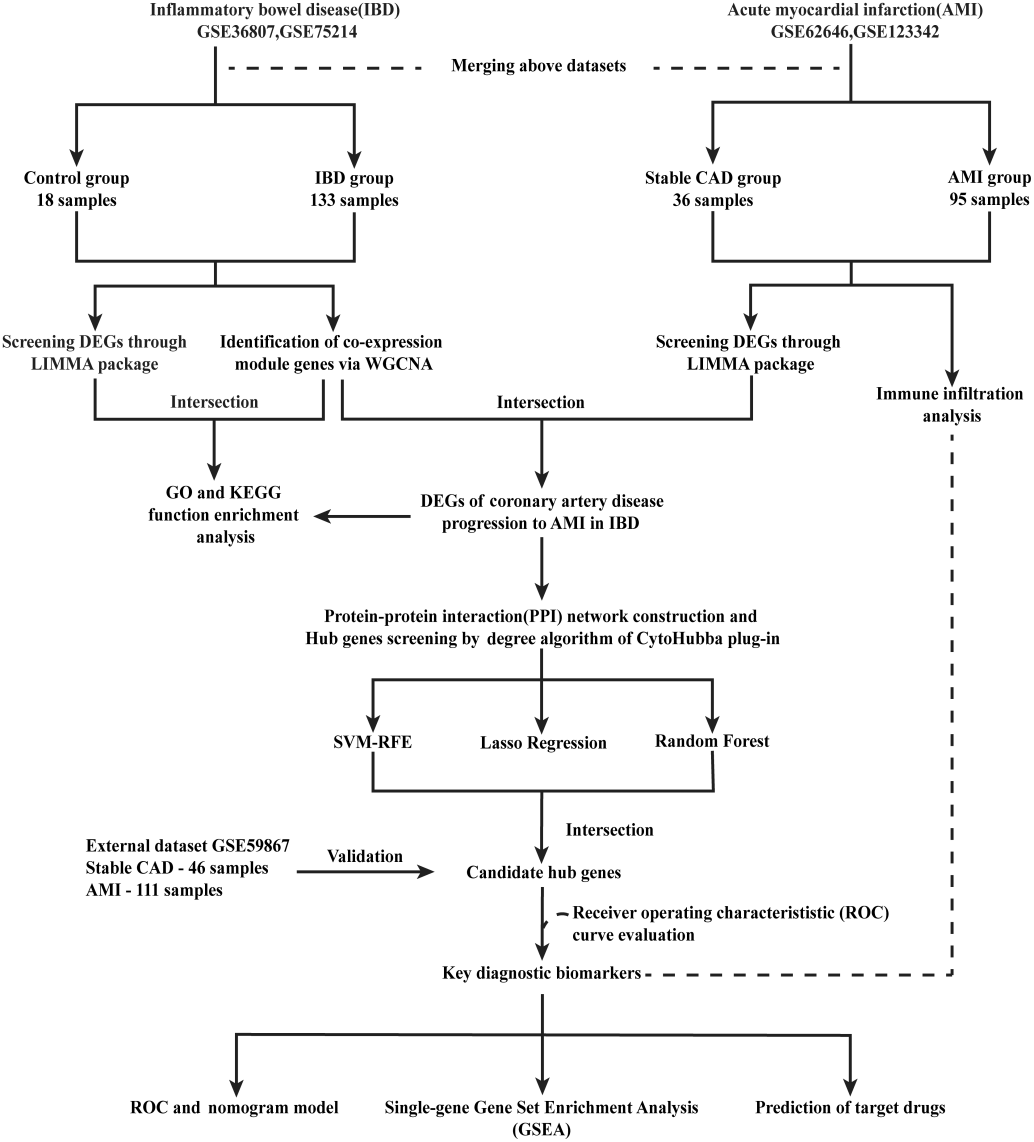
Flowchart of the study design.

The study design is well‐elucidated as a flowchart in Figure 1. We searched the Gene Expression Omnibus (GEO, https://www.ncbi.nlm.nih.gov/geo/) database for AMI and IBD related gene datasets by the utilization of the keywords ‘Acute Myocardial Infarction’ and ‘Inflammatory Bowel Disease’. The inclusion criteria were as follows: (1) Array‐based expression profiling of homo sapiens; (2) To ensure the accuracy of the results, the sample size for each group should be at least five. Finally, five accessible mRNA datasets (test datasets: GSE62646,^31^ GSE123342,^32^ GSE75214^33^ and GSE36807^34^ and validation dataset: GSE59867^35^) were included. Thorough information of abovementioned datasets is shown in Table 1, including the study sample size, tissues and corresponding annotation platform.

**TABLE 1:** Detailed information of expression profile datasets performed in the study.

		Sample size				
GSE series No.	Type	Stable CAD	AMI	Source tissue	Platform	Cohort
GSE62646	mRNA	14	28	PBMC	GPL6244	Test dataset
GSE123342	mRNA	22	67	Peripheral blood	GPL17586	Test dataset
GSE59867	mRNA	46	111	PBMC	GPL6244	Validation dataset

		Sample size				
GSE series No.	Type	Control	IBD	Source tissue	Platform	Cohort
GSE75214	mRNA	11	105	Colonic mucosa	GPL6244	Test dataset
GSE36807	mRNA	7	28	Intestinal biopsy	GPL570	Test dataset

*Note*: GSE36807 consists of 13 CD and 15 UC; GSE75214 consist of 8 CD and 97 UC.

Abbreviations: AMI, acute myocardial infarction; CAD, coronary heart disease; CD, Crohn's disease; IBD, inflammatory bowel disease; PBMC, peripheral monocytes; UC, ulcerative colitis.

### 2.2 Data processing and identification of differentially expressed genes

The raw data were extracted and background calibration, normalization and log2 transformation were performed with Robust Multi‐Array Average algorithm.^36^ Then the probe ID were converted into specific gene symbol based on platform annotation files. If multiple probes mapped to a common gene, the average value was calculated to determine its expression. Due to the batch effects among different microarray studies, empirical Bayesian methods were used to adjust the potential batch effects,^37^ generating a novel matrix of target‐disease AMI and IBD expression datasets (Figure S1), respectively. The ‘Limma’ package (v 3.40.6) was adopted to identify DEGs between Stable CAD and AMI by setting a cut‐off criteria of |Fold change (FC)| >1.3 and *p* < 0.05, as well as DEGs between IBD and controls with |FC| >2.0 and *p* < 0.05.

### 2.3 Crucial module identification via weighted gene co‐expression network analysis

It is well‐known that WGCNA is a systems biology strategy for investigating gene correlation patterns.^38^ We used WGCNA to construct a gene co‐expression network and identify crucial module genes significantly associated to IBD. In briefly, Median Absolute Deviation was calculated for each gene separately, and approximately half of the genes with the smallest values were deleted. Subsequently, a scale‐free co‐expression network was built via the goodSamplesGenes function, which capable of filtering DEG expression matrix to remove unqualified genes and samples. Then the weighted adjacency matrix was constructed based on the soft thresholding parameter (*β*) and transformed into a topological overlap matrix (TOM) to measure gene ratio and the corresponding dissimilarity (1‐TOM). Finally, modules with similar gene expression profiles were classified by hierarchical clustering and visualized using the dynamicTreeCut function. Average linkage hierarchical clustering was administrated according to the TOM‐based dissimilarity measure with a minimum size (Gene group) of 50 for the genes dendrogram. To further analyse these modules, a cut line was distributed to merge similar modules based on the dissimilarity of module eigengenes. Meanwhile, visual presentation on the eigengenes networks.

### 2.4 Functional enrichment analysis

Kyoto encyclopedia of genes and genomes (KEGG) and Gene Ontology (GO) are well‐established knowledge resources for comprehensive analysis of gene functions and biological linkage.^39^, ^40^ In order to further reveal the molecular mechanisms of CAD progression in patients with IBD. GO and KEGG pathway enrichment analyses of common genes (intersection of DEGs for AMI and module genes related to IBD) were conducted using ‘cluster profiler’ R package (v 4.6.2), with the outcomes were visualized in Sangerbox biomedical platform. (http://www.sangerbox.com). *p* < 0.05 is the statistically significant criteria.

### 2.5 Protein–protein interaction network

Protein‐protein interaction (PPI) network was constructed via STRING database (https://cn.string‐db.org; version 11.5), an online tool capable of identifying gene or protein interactions. In practice, 0.400 (medium confidence) was considered as the minimum required interaction score. To reduce potential interference and improve network confidence, genes that fail to interact with each other were removed. Subsequently, the data of the interacting genes was transferred to Cytoscape 3.9.1 software for visualization purposes. The top 20 genes were identified using the degree algorithm implemented in the CytoHubba, a plug‐in of Cytoscape.^41^

### 2.6 Multiple machine learning algorithms and candidate hub genes screening

To screen important diagnostic biomarkers for predicting CAD progression, three machine learning algorithms including Random Forest (RF), LASSO and SVM‐RFE were conducted on the identified AMI‐related DEGs with IBD. RF, a non‐linear classifier, can effectively highlight the key predictors by excavating the non‐linear interaction relationship between variables based on the decision trees.^42^ LASSO regression is classical computational learning method, which is characterized by variable selection and regularization for avoiding overfitting to improve liner‐model stability.^43^ Meanwhile, SVM‐RFE is applied to continuously perform iterative analysis on all genes, resulting in a gene ranking list according to characteristics importance.^44^ In R environment, ‘randomForest,’ ‘glmnet’ and ‘caret’ packages were employed to develop corresponding deep machine learning analyses. The intersection genes of RF, LASSO and SVM‐RFE were selected as crucial candidates for AMI prediction and diagnosis.

### 2.7 Receiver operating characteristics (ROC) curve and nomogram construction

First, the ROC curve was drawn to evaluate the diagnostic value of identified candidate biomarkers and to further screen key genes from candidates. The area under the curve (AUC) and 95% CI of candidate genes were calculated, AUC > 0.8 was considered the ideal key gene for nomogram construction using ‘rms’ package (v 6.7‐0). In briefly, calculating the total score by the summing all key genes score, and positioned to the point of probability axis to predict the AMI occurrence in IBD patients. Subsequently, the nomogram was also quantified with ROC to estimate the stability of the predictive model.

#### 2.7.1 Immune landscape analysis

CIBERSORT algorithm is a computational strategy capable of transforming the normalized gene expression matrix into the distribution of immune infiltration cells.^45^ Using the LM22 as a reference expression signature, the relative proportion of different immune cells between stable CAD and AMI groups was calculated via ‘CIBERSORT’ R package (v 0.1.0). Only samples with a CIBERSORT *p* ≤ 0.05 were retained for further analysis. Then the ‘ggplot2’ package (v 3.4.1) was employed to visualize the output results in bar plots. Additionally, we also analysed the correlation between the key biomarkers and infiltrating immune cells, as well as the relationship between immune cells.

#### 2.7.2 Single‐gene Gene Set Enrichment Analysis and prediction of target drug

Gene Set Enrichment Analysis (GSEA) for single‐gene is a bioinformatics approach that utilizes expression profiles to investigate the functions and signalling pathways associated with diagnostic biomarkers in certain biological process and diseases. In this study, we utilized the ‘GSEA’ r package to compute the correlation between each diagnostic biomarker and all other genes. Then, the genes were ranked in descending order regarding their correlation values. The ranked genes were designated as the test gene set, and the predefined gene sets consisted of KEGG signalling pathways. Consequently, we evaluated the enrichment level of corresponding test gene set in the KEGG signalling pathways to further understand its potential physiological and pathological mechanisms. Cut‐off criteria with |normalized enrichment score (NES)| >0.5, adjusted *p* < 0.01 and *q* < 0.05. Furthermore, based on the identified biomarkers, the ‘Enrichr’ online database (https://maayanlab.cloud/Enrichr/) was employed to predict potential target‐drugs for the management of IBD patients with a high risk of CAD progression.

### 2.8 Statistical analysis

In the study, statistical analysis was performed by SPSS (IBM Corporation, Armonk, NY, USA; Version 25.0) and R (version 4.3.0; win) software. Student's *t*‐test or Wilcoxon rank‐sum test were employed to investigate the differences of infiltrating immune cells in two clusters via GraphPad Prism (San Diego, California, USA; Version 9.4.0). Two‐sided *p* < 0.05 is considered statistically significant.

## 3 RESULTS

### 3.1 Identification of DEGs

**FIGURE 2:**
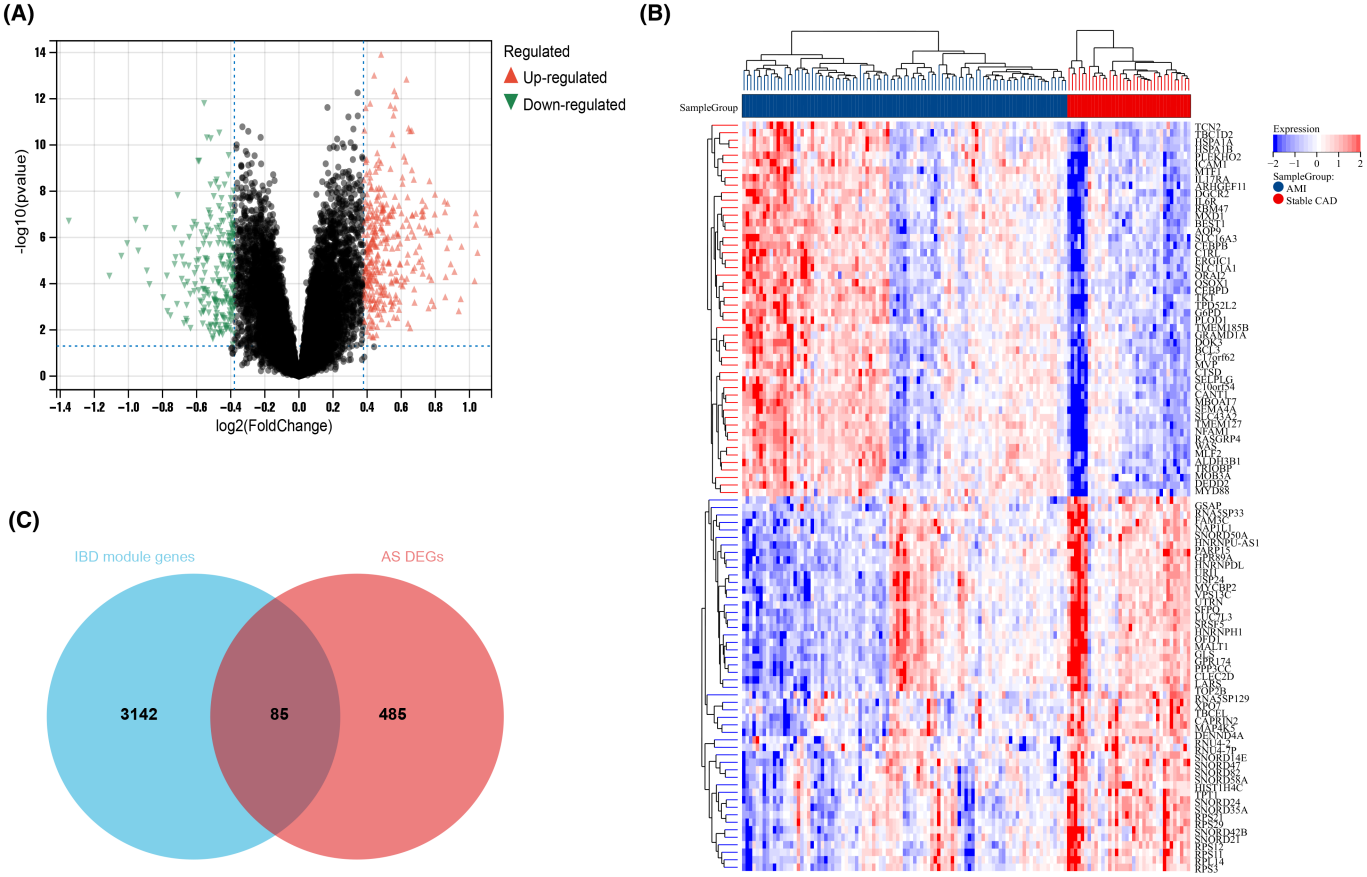
Identification of the DEGs associated to CAD progression between Stable CAD and AMI groups from the integrated dataset(GSE62646, GSE123342). (A) The volcano plot of all genes, with red and green triangles indicate 340 up‐ and 230 down‐regulated DEGs, respectively. (B) The heatmap plot of 50 up‐ and down‐regulated DEGs, red and blue grids indicate up‐ and down‐regulated DEGs, respectively. (C) The intersection of WGCNA significant modules genes related to IBD and above‐identified DEGs of stable CAD progression to AMI, be visualized with Venn diagram. AMI, acute myocardial infarction; CAD, coronary artery disease; IBD, inflammatory bowel disease; DEGs, differentially expressed genes; WGCNA, Weighted Gene Co‐expression Network Analysis. |FC| >1.3 and *p* < 0.05.

As shown in Figure 2A,B, a total of 570 DEGs were screened between AMI and Stable CAD groups of the AMI combined dataset by using the ‘Limma’ package, where 340 were upregulated and 230 were downregulated. As for IBD, there were 430 DEGs (283 upregulated and 147 downregulated; criteria: |FC| >2, *p* < 0.05) in IBD compared to healthy controls (Figure S2A,B).

### 3.2 Key module selection related to IBD by WGCNA

**FIGURE 3:**
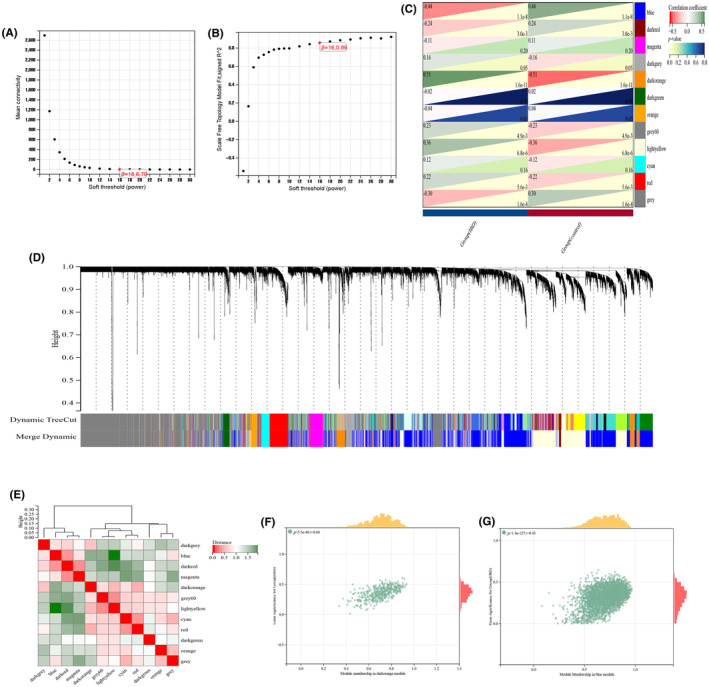
Identification of related modules associated with inflammatory bowel disease (IBD) by weighted gene co‐expression network analysis (WGCNA). (A, B) *β* = 18 was regarded the best soft‐power value according to scale independence and average connectivity. (C) The heatmap to visualize the correlation between the different modules in the non‐IBD control and IBD groups. (D) A gene clustering tree with multiple divided modules. Different clusters were attached with different colours. (E) Heatmap of eigengene adjacency. (F, G) The scatter plots of module membership and gene significance for IBD.

The WGCNA analysis was well‐described in our previous study.^46^ In briefly, Figure 3A,B indicated that the best soft‐power value for IBD merged dataset (GSE75214 + GSE36807) was 16 according to scale independence and average connectivity. After modules merging, a total of 12 co‐expression modules were recognized, each corresponding to one colour (Figure 3D,E). The correlation analysis between modules and features showed that dark‐orange (393 genes, correlation coefficient (CC) = 0.51, *p* < 0.0001) and blue modules (2834 genes, CC = −0.44, *p* < 0.0001) had the most positive and negative correlation in IBD (Figure 3C), respectively. Moreover, the correlation of module membership in dark‐orange (*r* = 0.60) and gene significance for IBD samples was observed, as well as blue module membership (*r* = 0.43) (Figure 3F, G).

### 3.3 Functional enrichment analysis and identification of DEGs via PPI network

**FIGURE 4:**
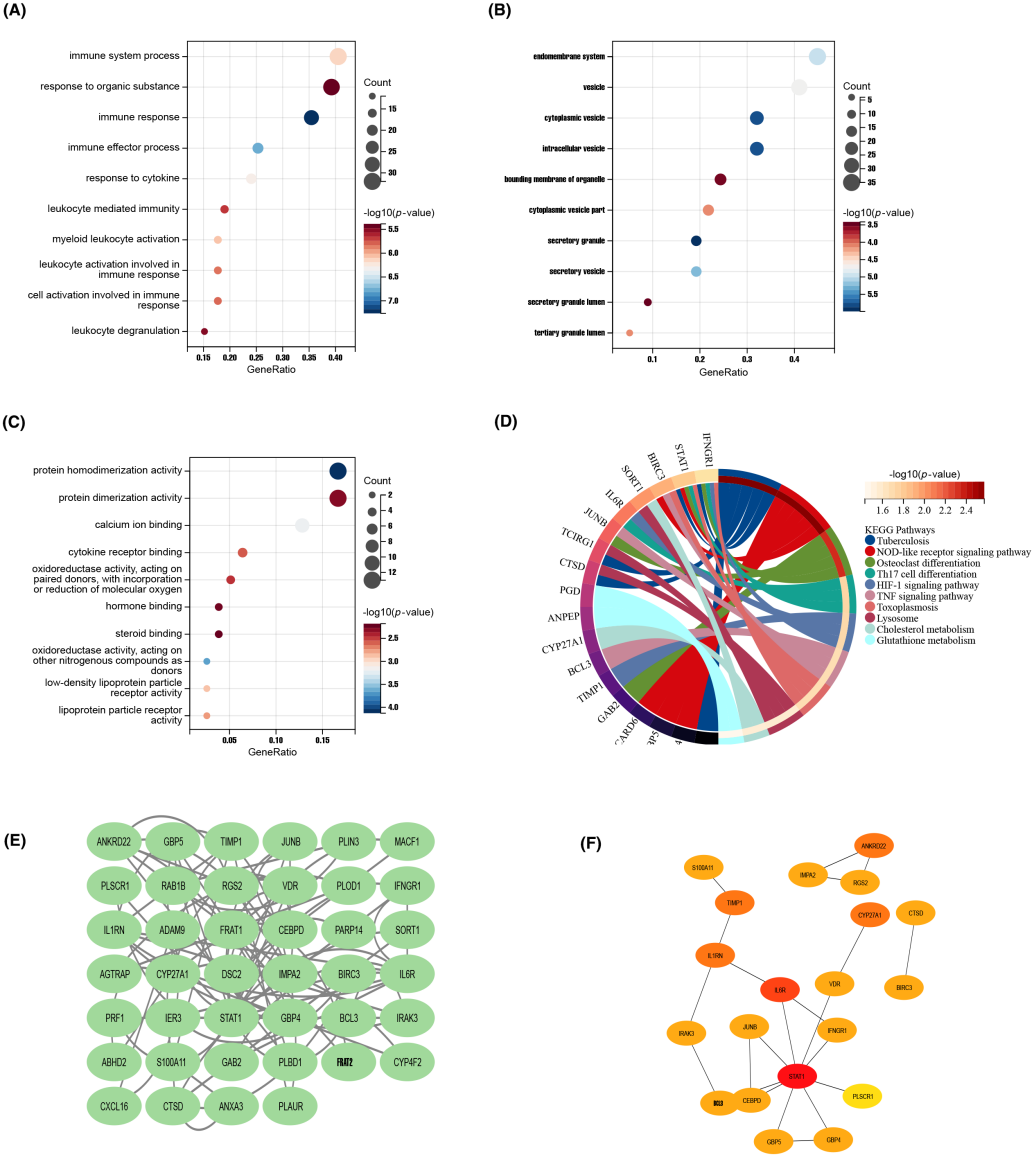
Functional enrichment analysis of IBD‐related DEGs in AMI and identification of candidate hub genes via protein‐protein interaction (PPI) network. (A–C) Gene Ontology (GO) functional analysis of IBD‐related DEGs in AMI, consists of biological function, cellular component and molecular function. The *X*‐axis and *Y*‐axis represent gene ratio and GO terms, respectively; The colour of the circle represents significance, and the size indicates the number of enrichment genes. (D) KEGG pathway analysis of IBD‐related DEGs in AMI. (E, F) The PPI network of 40 interacted genes, and top 20 were screened out by ‘degree’ algorithm and visualized as a network in Cytoscape. KEGG, Kyoto Encyclopedia of Genes and Genomes.

A total of 85 common genes were identified at the intersection of IBD‐related significant modules and DEGs of revealing CAD progression (Figure 2C). In order to explore the pathogenic mechanisms underlying molecules, GO and KEGG function enrichment analysis was conducted on the shared genes. The GO analysis suggested that those DEGs were primarily enriched in several terms: (1) biological process (BP), including immune response, immune effector process, response to cytokine and immune systems process; (2) cellular component (CC), including cytoplasmic/intracellular/secretory vesicle, secretory granule; and (3) molecular function (MF), including protein homodimerization activity (Figure 4A–C). The KEGG pathway enrichment analysis showed these DEGs were mainly enriched in NOD‐like receptor signalling pathway, Th17 cell differentiation and TNF/HIF‐1 signalling pathways (Figure 4D). The results of function enrichment annotation indicated that IBD‐related module genes may participate in the progression of coronary atherosclerotic lesion from the aspects of immune and inflammatory response. Similarly, function enrichment analysis of the IBD‐related module DEGs (the intersection of IBD key module gens and identified DEGs between IBD individuals with controls) was elucidated in Figure S3.

To further detect hub genes that play a key role in CAD progression with IBD disorder. After removing 45 non‐interactive genes, PPI network was constructed based on the remaining 40 node gens which interact with each other (Figure 4E). Subsequently, degree method was employed to quantify node genes and ranked, the top 20 were screened out and visualized as a network presented in Figure 4F. Detailed information of the 20 genes is listed in Table S1.

### 3.4 Identification of candidate hub genes via multi‐machine learning methods

**FIGURE 5:**
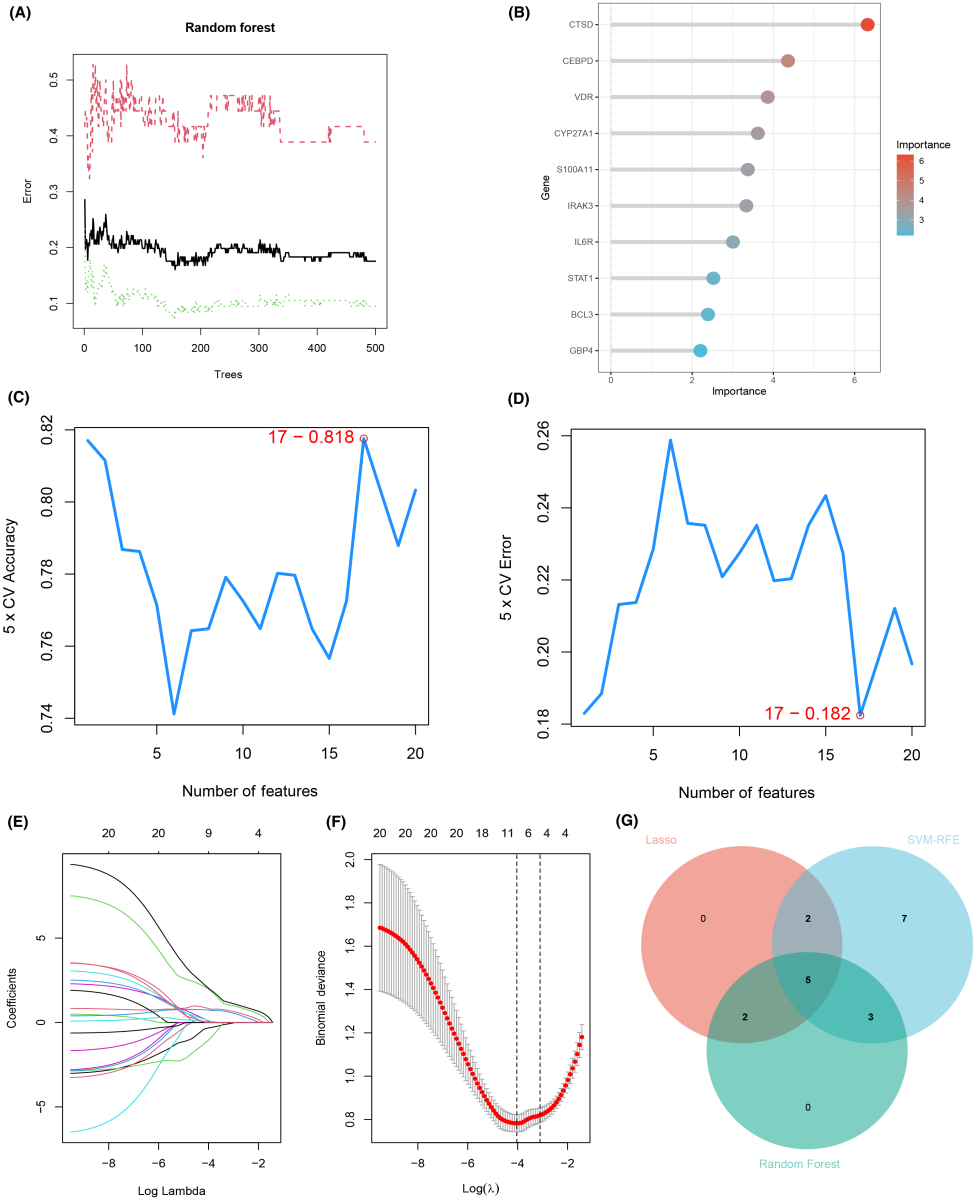
Selection of candidate hub DEGs for coronary atherosclerotic lesions progression using multi‐machine learning algorithms. (A, B) Gene selection via RF computational strategy. The top 10 genes listed in accordance with importance score. (C, D) SVM‐RFE algorithm was used to screen the significant feature genes out from 20 candidates. Seventeen genes (maximal accuracy = 0.818, minimal RMSE = 0.182) ultimately were recognized as the optimal feature genes. (E, F) Features screening in the LASSO regression model based on 10‐fold cross‐validation, nine genes corresponding to the lowest point of the curve were considered the most superior biomarkers for the forecast of CAD progression in patients with IBD. (G) The intersection of RF, SVM‐RFE and LASSO regression was performed and visualized with Venn plot, resulting in five hub genes (CTSD, CEBPD, CYP27A1, STAT1 and S100A11) for further ROC evaluation and nomogram construction. RF, random forest; SVM‐RFE, support vector machine‐recursive feature elimination; LASSO, least absolute shrinkage and selection operator.

RF, SVM‐RFE and LASSO regression machine learning strategies were used to screen candidate hub biomarkers for the next step of analysis. The RF algorithm was applied to select top 10 genes in accordance with importance score (Figure 5A, B). In the SVM‐RFE, the top 17 genes had the lowest error and highest accuracy in predicting the progression of CAD with IBD (Figure 5C, D). As for LASSO regression, we observed that nine genes can achieve the lowest binomial deviance in the curve(Figure 5E, F).Ultimately, intersection on the results of three machine learning algorithms and visualized through Venn diagram. Five candidate hub genes (CTSD, CEBPD, CYP27A1, STAT1 and S100A11) were obtained for further ROC assessment and nomogram construction.

### 3.5 Evaluation and determination of key biomarkers and the nomogram construction

**FIGURE 6:**
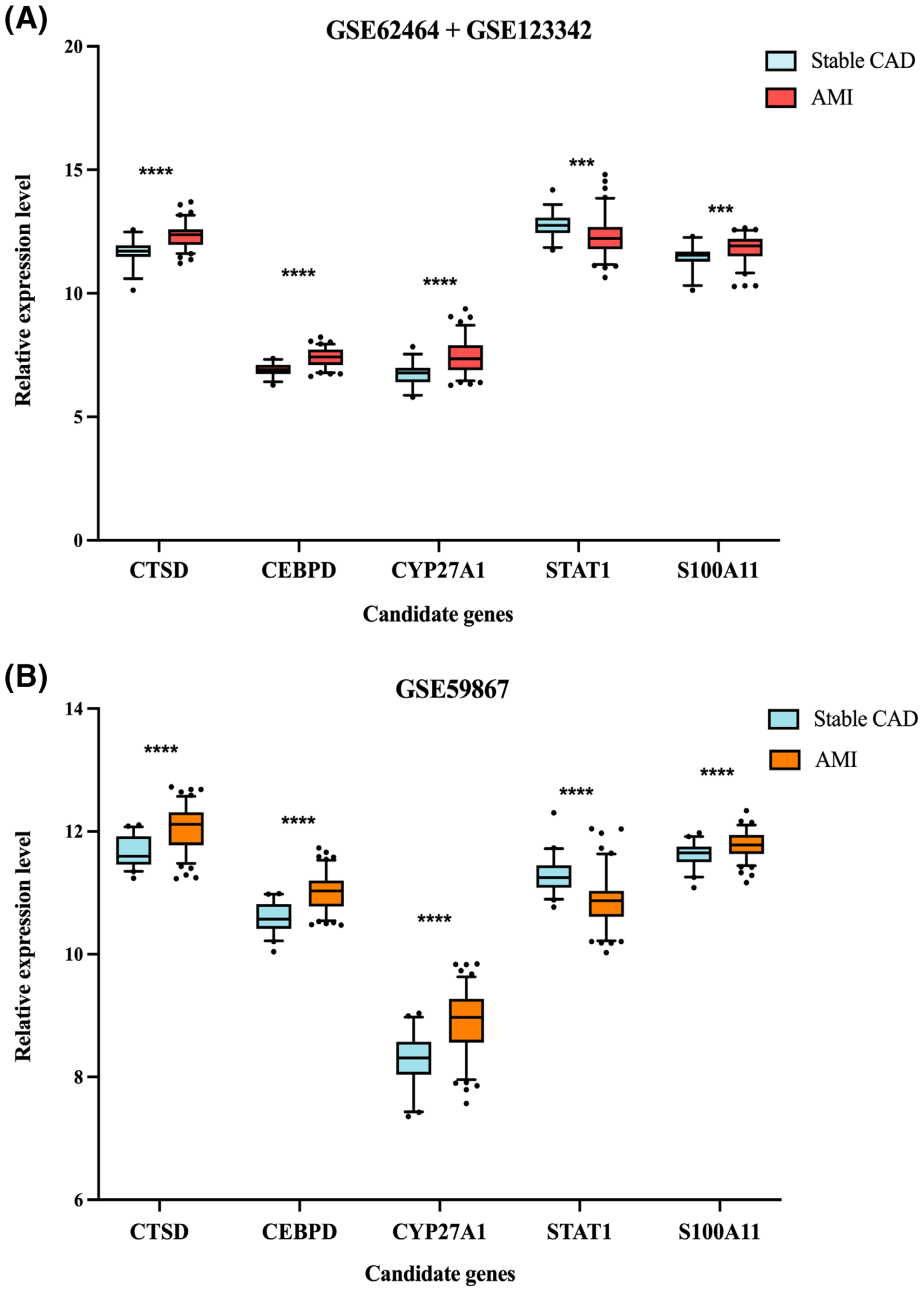
The relative expression levels on the five candidate genes (CTSD, CEBPD, CYP27A1, STATA and S100A11) between stable CAD and AMI groups. (A) Comparison of the candidate genes expression levels in test dataset (GSE62464 + GSE123342) conducted by Student's *t*‐test or Wilcoxon rank‐sum test. (B) Comparison of the candidate genes expression levels in validation dataset (GSE59867). AMI, acute myocardial infarction; CAD, coronary artery disease. ***p < 0.001; ****p < 0.0001.

**FIGURE 7:**
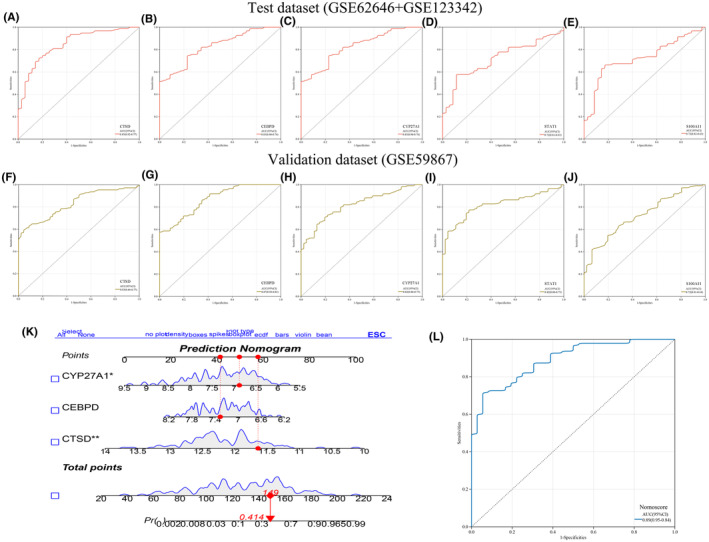
Evaluation and review of biomarker predictive capability and nomogram construction. (A–E) The ROC curves of five candidate hub biomarkers (CTSD, CEBPD, CYP27A1, STAT1 and S100A11) in the integrated validation set (GSE62464 + GSE123342). (F–J) The ROC curves of the five candidate hub biomarkers in the GSE59867 test set. (K) The nomogram model was constructed based on a set of five genes (CTSD, CEBPD, CYP27A1). (L) The ROC diagnostic curve for nomogram. ROC, receiver operating characteristic.

The relative expression levels on the five candidate biomarkers of stable CAD versus AMI groups showed the same trend in the test and validation datasets, shown in Figure 6A,B. To assess the diagnostic capability of five candidate hub genes for predicting stable CAD development into AMI, ROC curves were constructed according to individual characteristic and the expression of each gene. AUC and the corresponding 95% CI for each gene was determined in test‐ and validation dataset, respectively. In the test set, CTSD (AUC 0.85, 95% CI 0.77–0.92), CEBPD (AUC 0.83, 95% CI 0.76–0.90), CYP27A1 (AUC 0.81, 95% CI 0.73–0.88), STAT1 (AUC 0.72, 95% CI 0.63–0.81), S100A11 (AUC 0.72, 95% CI 0.63–0.82) (Figure 7A–E). With regard to GSE59867 validation set, the results were CTSD (AUC 0.83, 95% CI 0.77–0.89), CEBPD (AUC 0.87, 95% CI 0.81–0.92), CYP27A1 (AUC 0.82, 95% CI 0.75–0.88), STAT1 (AUC 0.82, 95% CI 0.75–0.88), S100A11 (AUC 0.72, 95% CI 0.64–0.81) (Figure 7F–J), respectively. Ultimately, three genes (CTSD, CEBPD and CYP27A1) were selected to nomogram construction in terms of inclusion criteria (AUC > 0.8). In the nomogram (Figure 7K), quantifying the relative expression of each gene by marks, the total score was contributed to assess the risk of coronary atherosclerotic lesions progression in IBD patients. Moreover, the AUC was 0.89 (95% CI 0.84–0.95), revealing the superiority of this nomogram performance (Figure 7L).

### 3.6 Single‐gene GSEA

**FIGURE 8:**
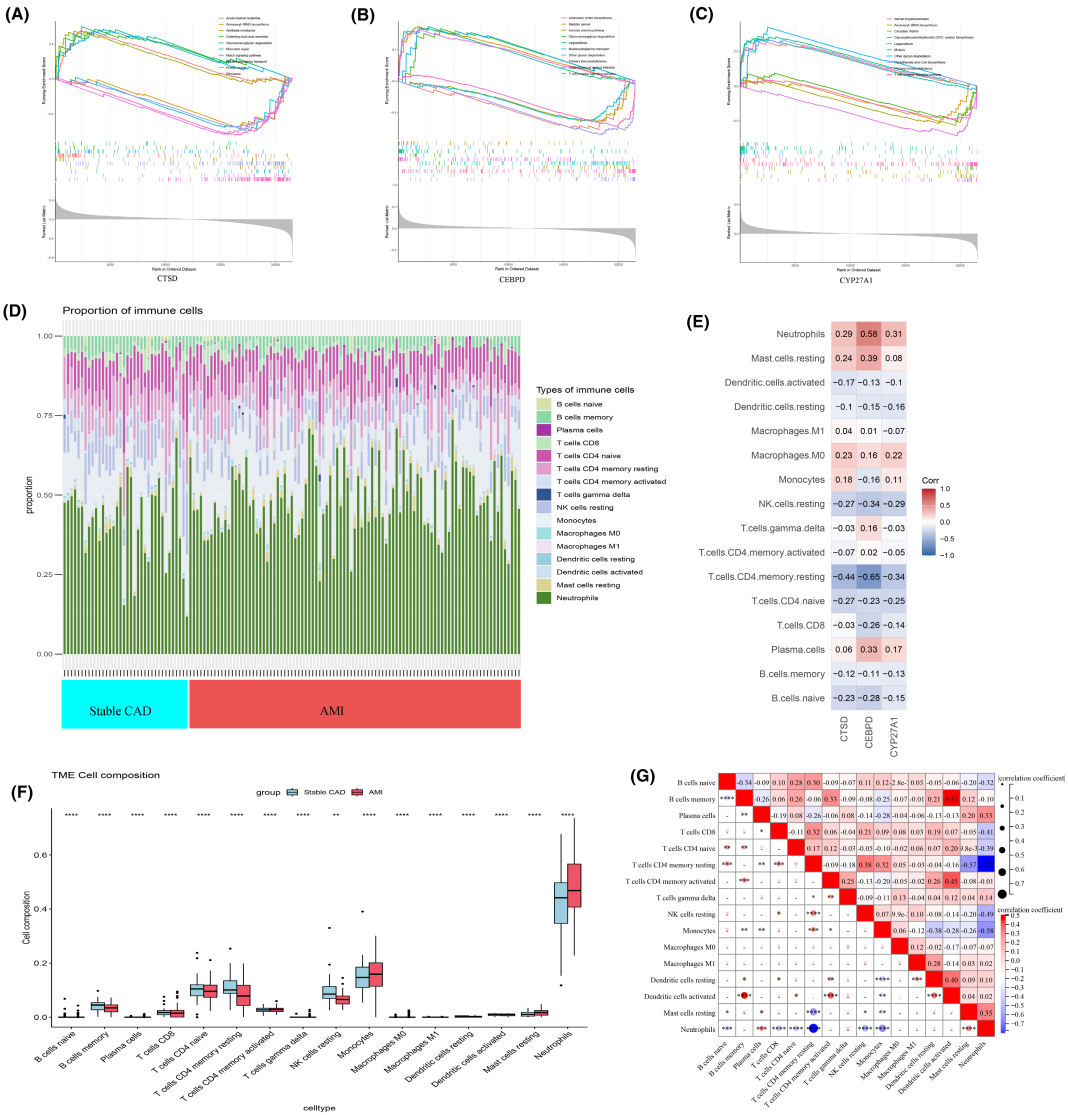
Single‐gene Gene Set Enrichment Analysis (GSEA) and Immune infiltration landscape. (A–C) Results of single‐gene GSEA, the lines in each figure indicate the top 10 enriched items: (A) single‐gene GSEA of CTSD; (B) single‐gene GSEA of CEBPD; (C) single‐gene GSEA of CYP27A1. (D) The stacked plot displaying the relative proportion of 16 immune cells in different samples from stable CAD and AMI groups. (E) The correlated heatmap between three key genes and immune cells. (F) The box plot comparing the expression of immune cells between stable CAD and AMI. (G) The heatmap showing the correlation between different immune cells in the pathogenesis of AMI. *p < 0.05; **p < 0.01; ***p < 0.001; ****p < 0.0001. AMI, acute myocardial infarction; CAD, coronary artery disease.

Herein, we performed GSEA on CTSD, CEBPD and CYP27A1 to explore the potential KEGG pathways of these genes which involved in CAD progression. Ten significant items of each gene were exhibited in Figure 8A–C. The results suggested that the three genes might be involved in the pathogen invasion, metabolism, immune and inflammation responses in the course of coronary atherosclerosis with IBD. For instance, CTSD is involved in the pathways of ‘Notch signaling pathway,’ CEBPD and CYP27A1 simultaneously mediate ‘T cell receptor signaling pathway,’ ‘Primary immunodeficiency’ and ‘bacterial infection’ signal pathways. Additionally, all genes were correlated with the metabolism of glycan. The single‐gene GSEA results of test dataset (GSE62646 + GSE123342) were presented in Figure S4.

### 3.7 Immune landscape analysis

As shown in Figure 8D, the relative percentage of 16 immune cells were visualized using stacked column plot. The rest six types of immune cells (T cells follicular helper, T cells regulatory (Tregs), NK cells activated, Macrophages M2, Mast cells activated, Eosinophils) were not detected in Cibersort algorithm. Compared to stable CAD group, AMI has higher proportion of neutrophils, monocytes and mast cells resting, and lower proportion of T cells, B cells and NK cells resting(Figure 8F). The correlation heatmap between genes and immune cells displayed that both CTSD, CEBPD and CYP27A1 were negatively correlated with CD4^+^ T cells and positively correlated with neutrophils (Figure 8E). Meanwhile, a significant negative relationship of neutrophils and CD4^+^ T cells was observed, especially for CD4^+^ memory resting T cells (correlation coefficient = −0.77) (Figure 8G). These findings uncovered a potential crosstalk mechanism between the biomarkers and neutrophils with CD4^+^ T cells during the CAD progression in patients with IBD.

### 3.8 Prediction of target drugs

Ten drugs were predicted to be IBD‐related drugs, which may become the potential therapies for AMI (Table S2). CTSD is the biomarker most associated with these drugs, indicating the enormous latent capacity of this biomarker as a drug target for AMI.

## 4 DISCUSSION

It is well‐established that most AMIs occur in the setting of the complete coronary occlusion caused by erosion and/or sudden rupture of atherosclerotic plaque accompanied by secondary thrombosis.^47^ The unstable property of plaques is regarded to be responsible for driving CAD progression towards to AMI.^48^ In addition to the continuous deposition of cholesterol, fat substances, fibrin and other metabolic wastes, the progressive inflammation induced by infiltration of massive immune cells and cytokines plays a crucial role in the plaques beneath the arterial wall.^49^, ^50^ IBD is a chronic inflammatory disease with elevated serum inflammatory biomarkers, which capable of mediating systemic inflammation cascade responses, especially during active periods.^51^ There is increasing evidence revealing that IBD is the contributing factor to the progression of CAD, but the delicate crosstalk mechanisms underlying these two inflammation‐related diseases are ambiguous to date.

In our study, after a series of sifting and analysis, three immune‐ and inflammation‐associated genes (CTSD, CEBPD and CYP27A1) were identified for predicting the CAD progression in patients with IBD. To further test the diagnostic value of these biomarkers in clinical practice, we constructed ROC curves and a nomogram for quantitative assessment. Immunocyte infiltration analysis showed that both three genes were negatively correlated with CD4^+^ T cells and positively correlated with neutrophils. In addition, ‘Enrichr’ database was used to explore the upstream target drugs in AMI population based on the identified biomarkers.

CTSD gene provides unique instructions for encoding a protein called cathepsin D.^52^ Cathepsin D belongs to a member of lysosomal cathepsin family, which has been proved to participate in neoplasm,^53^ degenerative^54^ and chronic inflammatory diseases^55^, ^56^ in the light of proteolytic and destructive characteristics. It is general knowledge that the gut tract is the largest battleground of human immunity, the identification of intestinal macrophages (IMACs) as vital in keeping intestinal homeostasis.^57^ In IBD and other inflammatory lesions; however, IMACs were significantly associated with overexpressed CTSD mRNA and the encoded product cathepsin D.^58^ Menzel et al. revealed that inhibition of CTSD could contribute to an alleviation of dextran‐sulphate‐sodium induced colitis in mice and less weight loss in comparison to controls.^59^ An increased activation of cathepsin D in the intestinal mucosa of IBD patients might enhance apoptosis of intestinal epithelial cells, leading to the destruction of the intestinal barrier. This hypothesis has been verified in animal models.^60^ In addition to working inside cells, cathepsin D is also supposed to be secreted into the extracellular space for controlling different pathophysiological mechanisms.^61^ Cathepsin D and lysosomal acid lipase, released from monocyte‐derived macrophages, which caused hydrolytic modification of the low‐density lipoprotein, resulting in lipid accumulation and the generation of foam cells in atherosclerotic plaques.^62^ Mohammadpour et al. demonstrated that the serum cathepsin D concentration elevated with more severe stenosis and increased number of affected coronary artery.^63^ In the present study, the expression of CTSD was up‐regulated in AMI versus stable CAD patients with IBD, apparently suggesting that higher CTSD levels have an advanced CAD status.

CCAAT/enhancer binding protein delta (CEBPD) is an intronless gene encoding a protein known as basic leucine zipper (bZIP) transcription factor.^64^ According to the current insights, CEBPD serves as a positive modulator in the production of pro‐inflammation factors and activation of innate immune responses.^65^, ^66^ For instance, Hungness et al.^67^ found an increase of CEBPB‐ and CEBPD‐mediated IL‐6 production in the intestinal epithelial cells stimulated with IL‐1β. Liu et al.^68^ revealed that CEBPD enhanced macrophage phagocytosis in A. fumigatus infection by binding to the promoter region of Pentraxin 3. Although the level of inflammation response is regulated by the immune system, excessive inflammation caused by uncontrolled immunity is detrimental and lead to a cluster of autoimmune diseases, including Alzheimer disease,^69^ autoimmune encephalomyelitis^70^ and rheumatoid arthritis.^71^ Lai et al.^72^ found that CEBPD promoting atherosclerotic plaque development by inducing lipid accumulation in M1 macrophages. However, the potential regulatory mechanism of CEBPD on IBD is still unknown so far. Our study suggested that CEBPD expression in AMI patients was upregulated compared to stable CAD, implying excessive inflammation and immune dysfunction might exert pivotal function during CAD progression.

Sterol 27‐hydroxylase (CYP27A1) is a key rate‐limiting enzyme that catalyses the synthesis of bile acids in the liver, as well as functions in various tissues.^73^ Since the efflux of bile acids is the major form for reducing cholesterol stress in the body, this protein is crucial for the maintenance of the overall cholesterol homeostasis.^74^ It is reported that mutations in CYP27A1 gene account for cerebrospinal xanthomatosis, a rare lipid storage disorder characterized by atherosclerosis and neurological progressive dysfunction.^75^ Babiker et al. announced their discovery that elimination of cholesterol in macrophages via sterol 27‐hydroxylase, serving as a supplement to HDL‐mediated cholesterol reverse transport for the plaque alleviation.^76^ Besides, lipid metabolism plays a crucial part in modulating innate and adaptive immune pathways.^77^ Nuclear receptors, such as PPARs and LXRs, are activated by sensing the alteration of non‐esterified fatty acids and cholesterol metabolites to relieve type 2 diabetes‐mediated vascular inflammatory damage.^78^ The present study identified that CYP27A1 was down‐regulated in IBD patients with impaired transformation of bile acid and aberrant gut microbiota. Conversely, we observed a higher CYP27A1 expression in AMI compared to stable CAD, implying sterol‐27 hydroxylase acts as protective factor against atherosclerosis.

Aberrant and persistent immune and inflammatory responses are regarded as the feature‐hallmarks of IBD.^79^ During the progression of coronary atherosclerotic lesions, the plaque growth has been accompanied by immunity and inflammation until occurrence of AMI.^80^ As everyone knows, immune dysfunction induces systemic progressive inflammation which contributes to the risk of CAD proceeding to AMI in IBD patients.^81^, ^82^ GO and KEGG analysis showed that enriched results mainly refer to immune and inflammation‐related items and pathways, including immune response, immune effector process, and response to cytokine and immune systems process in GO, and NOD‐like receptor signalling pathway, Th17 cell differentiation and TNF/HIF‐1 signalling pathways in KEGG. It is well‐known that the recruitment and activation of immune cells, release of cytokines and production of inflammatory mediators were implicated in the atherosclerotic plaque formation and destabilization.^83^, ^84^ The presence of IBD, with its chronic inflammatory state, may contribute to systemic immune activation and exacerbate the inflammatory processes involved in atherosclerosis, potentially leading to an increased risk of coronary lesion progression. Moreover, we used the cibersort algorithm to analyse the landscape of 16 immune infiltrating cells from AMI blood samples. The results indicated that AMI has a relatively higher proportion of neutrophils, as well as a lower proportion of T and B cells compared to stable CAD samples. Myocardial infarction causes aseptic inflammation, which accompanied by the recruitment and activation of innate and adaptive immune cells.^85^ Within 24 h post‐AMI, neutrophils would be immediately recruited to infiltrate the infarcted myocardium and reach a peak at 3 days, followed by high abundance of M1 and M2 macrophages.^85^, ^86^ However, T and B cells principally play a role in the advanced stage of myocardial infarction. For example, Hee et al. announced that CD8^+^ CD57^+^ T cells was associated with increased cardiovascular mortality after AMI.^87^ Xu et al. suggested implantation of bone marrow‐derived B cells significantly diminished the myocardial infarction area in AMI‐mice.^88^

Neutrophils^89^ are primarily involved in the innate immune response and CD4^+^ T cells^90^ play a crucial role in the adaptive immune responses. The results of immune cell infiltration indicated that the expression level of CTSD, CEBPD and CYP27A1 are associated with the increased neutrophils and decreased CD4^+^ T cells. It suggested that both innate and adaptive immune responses are involved in the progression of CAD in patients with IBD, which is consistent with the facts.^91^, ^92^ Therefore, understanding the crosstalk between these identified biomarkers and immune cells, specifically neutrophils and CD4^+^ T cells, provides insights into the underlying mechanisms of CAD progression in IBD patients. Despite the number of related‐study is limited, it provides a novel potential target for the treatment of AMI with IBD.

Although the current research offered several inspired insights, there were still some limitations. First, there is an absence of sample clinical information related to central disease in the included GEO projects, including previous medicine, family history, and some basic clinical characteristics, which lead to bias on the results. Second, the validation set only cover stable CAD and AMI patients, without involving IBD simultaneously. Third, whether the key biomarkers identified of this study can be applied in clinical practice requires further validation from bench to bedside, though the nomogram has a high diagnostic value (AUC = 0.89). Thus, shortly afterwards we need to conduct more relevant studies personalized to CTSD, CEBPD and CYP27A1.

## 5 CONCLUSION

Our study identified three key biomarkers through the application of bioinformatics strategy and multiple machine learning algorithms, including CTSD, CEBPD and CYP27A1 which capable of predicting the risk of CAD proceeding to AMI in patients with IBD. Unusual immune landscape indicated that neutrophils and T lymphocytes play a crucial role in the occurrence and prognosis of AMI. These findings provide a great prospect for us to predict and investigate the progression of coronary atherosclerotic lesions.

## AUTHOR CONTRIBUTIONS

**Xiaoqi Tang:** Conceptualization (equal); data curation (lead); formal analysis (lead); investigation (lead); methodology (lead); resources (equal); software (equal); validation (equal); visualization (equal); writing – original draft (equal); writing – review and editing (equal). **Yufei Zhou:** Conceptualization (equal); methodology (equal); validation (equal); writing – original draft (equal); writing – review and editing (equal). **Zhuolin Chen:** Writing – review and editing (lead). **Chunjiang Liu:** Conceptualization (equal); formal analysis (equal); methodology (equal); project administration (equal); visualization (equal); writing – original draft (lead); writing – review and editing (lead). **Zhifeng Wu:** Data curation (equal); formal analysis (equal); investigation (equal); visualization (equal). **Yue Zhou:** Conceptualization (equal); data curation (equal); investigation (equal); methodology (equal); writing – original draft (equal); writing – review and editing (equal). **Fan Zhang:** Data curation (equal); formal analysis (equal); investigation (equal); methodology (equal); software (equal); visualization (equal); writing – original draft (equal). **Xuanyuan Lu:** Conceptualization (equal); funding acquisition (equal); writing – review and editing (equal). **Liming Tang:** Conceptualization (lead); funding acquisition (lead); project administration (lead); supervision (lead); writing – original draft (equal); writing – review and editing (lead).

## FUNDING INFORMATION

The current study was supported by the Shaoxing Scientific and Technological Program of Zhejiang Province (2022KY032) and the Natural Science Foundation of Zhejiang Province (LGF22H060031).

## CONFLICT OF INTEREST STATEMENT

The authors declare no conflicts of interest related to this study.

## Supporting information


Data S1:

## Data Availability

The datasets used (GSE62646, GSE123342, GSE75214, GSE36807 and GSE59867) in the study can be acquired from GEO database. The data and corresponding processing procedures during the present study are available from the corresponding author (Liming Tang) upon reasonable requests.
