# Supplementary material for: Identification of key biomarkers for predicting CAD progression in inflammatory bowel disease via machine‐learning and bioinformatics strategies
Source: J Cell Mol Med. 2024 Mar 7;28(6):e18175. doi: 10.1111/jcmm.18175 (PMC10919158; doi:10.1111/jcmm.18175)
Supplement: Supplementary file 1 — Data S1: [file JCMM-28-e18175-s001.docx]

**Supplementary material**

Figure

**
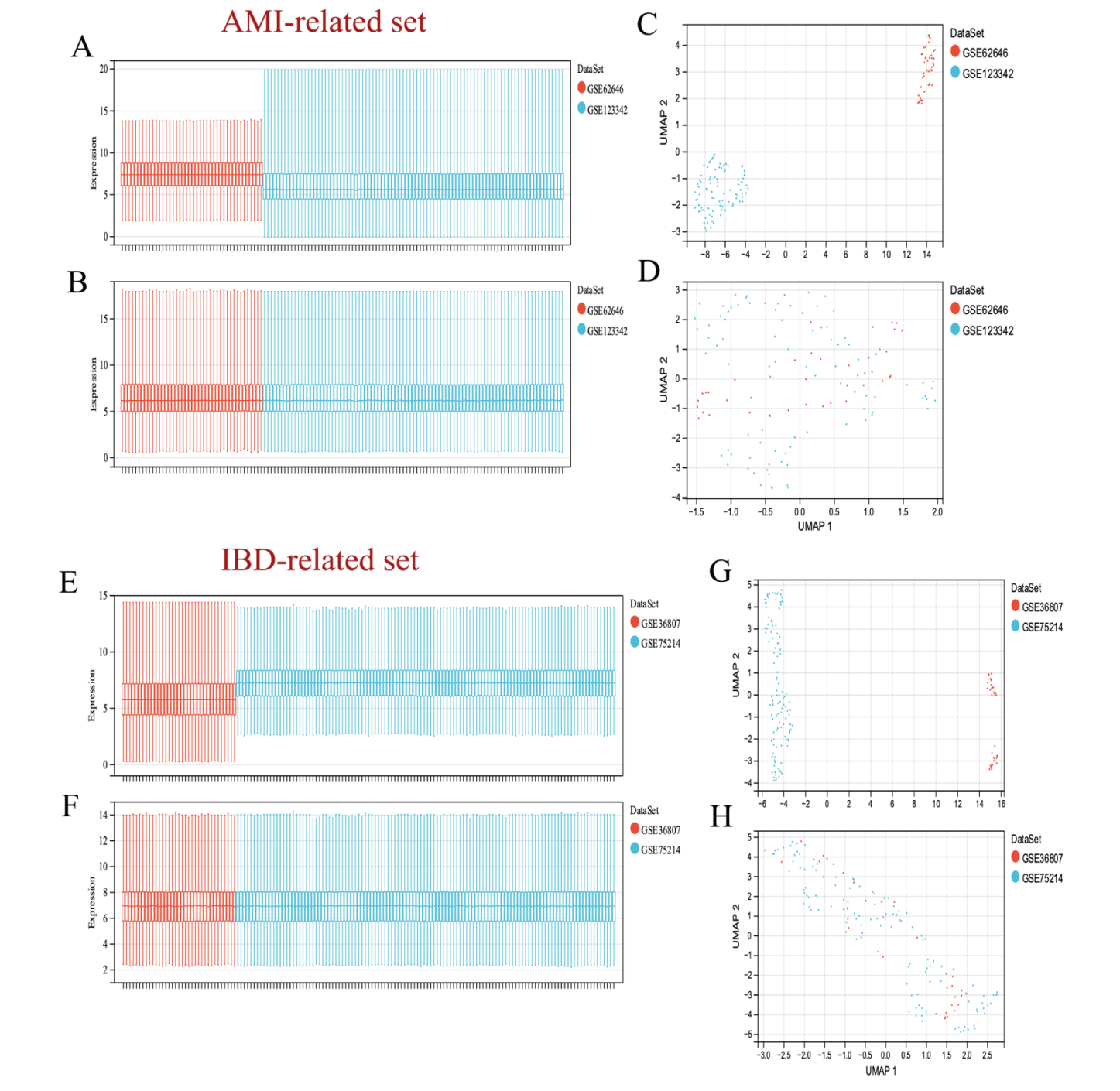
**

**Supplementary Figure 1.** Data preprocessing - Box plot and UMAP algorithm were performed to remove batch effects of GSE62646, GSE123342, GSE36807, and GSE75214. **(A, C)** before batch correction and **(B, D)** after batch correction for AMI-related sets. **(E, G)** before batch correction and **(F, H)** after batch correction for IBD-related sets. AMI, Acute myocardial infarction; IBD, Inflammatory bowel disease; UMAP, Uniform Manifold Approximation and Projection.


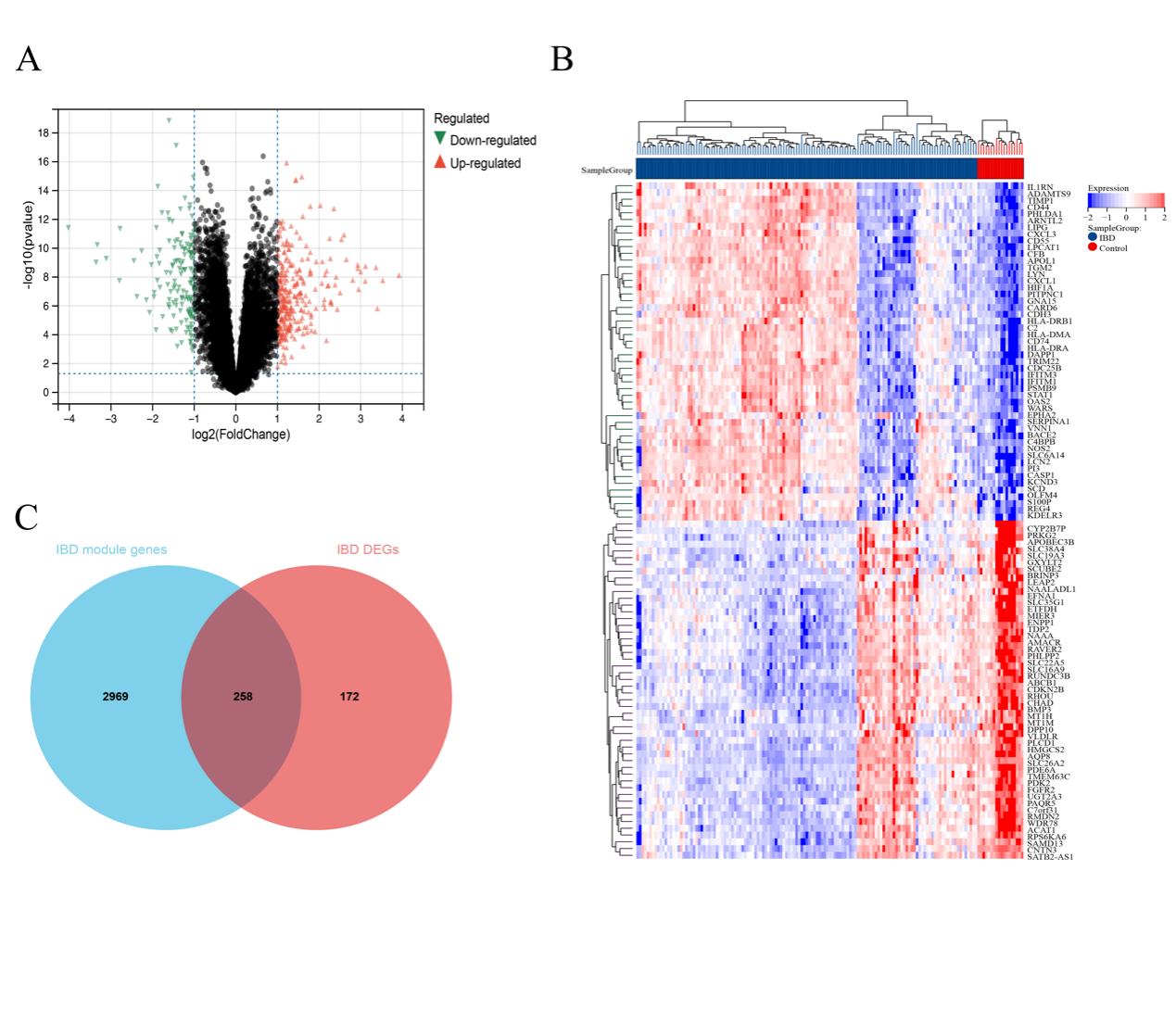


**Supplementary Figure 2.** Identification of the DEGs related to between IBD patients and healthy controls from the combined dataset(GSE75214, GSE36807). **(A)** The volcano plot of all genes, with red and green triangles indicate 283 up- and 147 down-regulated DEGs, respectively. (B) The heatmap plot of 50 up- and down-regulated DEGs, red and blue grids indicate up- and down-regulated DEGs, respectively. (C) The intersection of WGCNA modules genes related to IBD and DEGs of IBD compared to healthy controls, be visualized with Veen diagram. IBD, Inflammatory bowel disease; DEGs, differentially expressed genes; WGCNA, Weighted Gene Co-expression Network Analysis. **|Fold change| > 2.0** and *P*-value < 0.05.


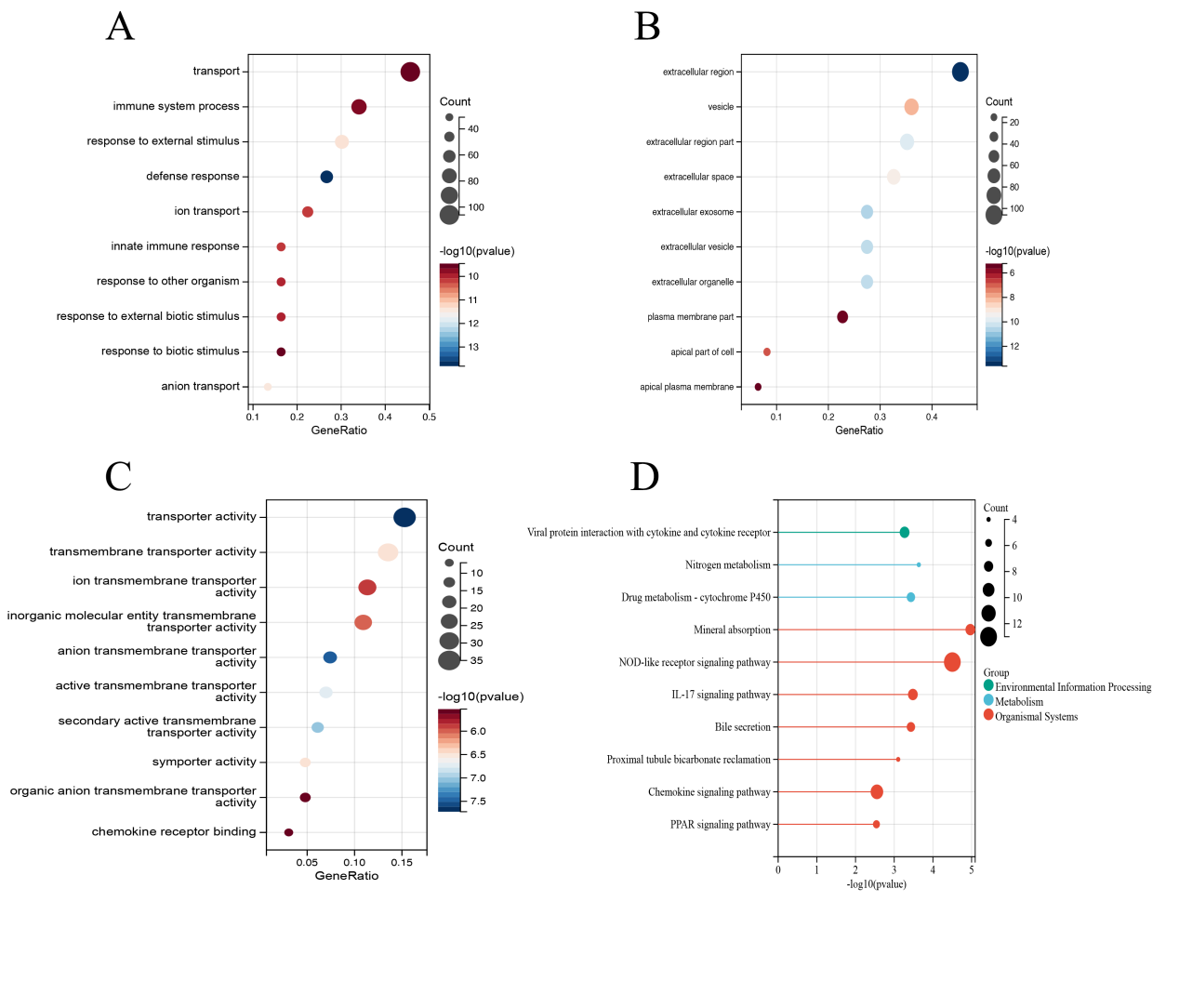


**Supplementary Figure 3.** Functional enrichment analysis of DEGs related to IBD module. **(A-C)** Gene Ontology (GO) functional analysis of DEGs related to IBD module, consisting of biological function, cellular component and molecular function. The X-axis and Y-axis represent gene ratio and GO terms, respectively; The color of the circle represents significance, and the size indicates the number of enrichment genes. **(D)** KEGG pathway analysis of DEGs related to IBD module. DEGs, differentially expressed genes; IBD, Inflammatory bowel disease.


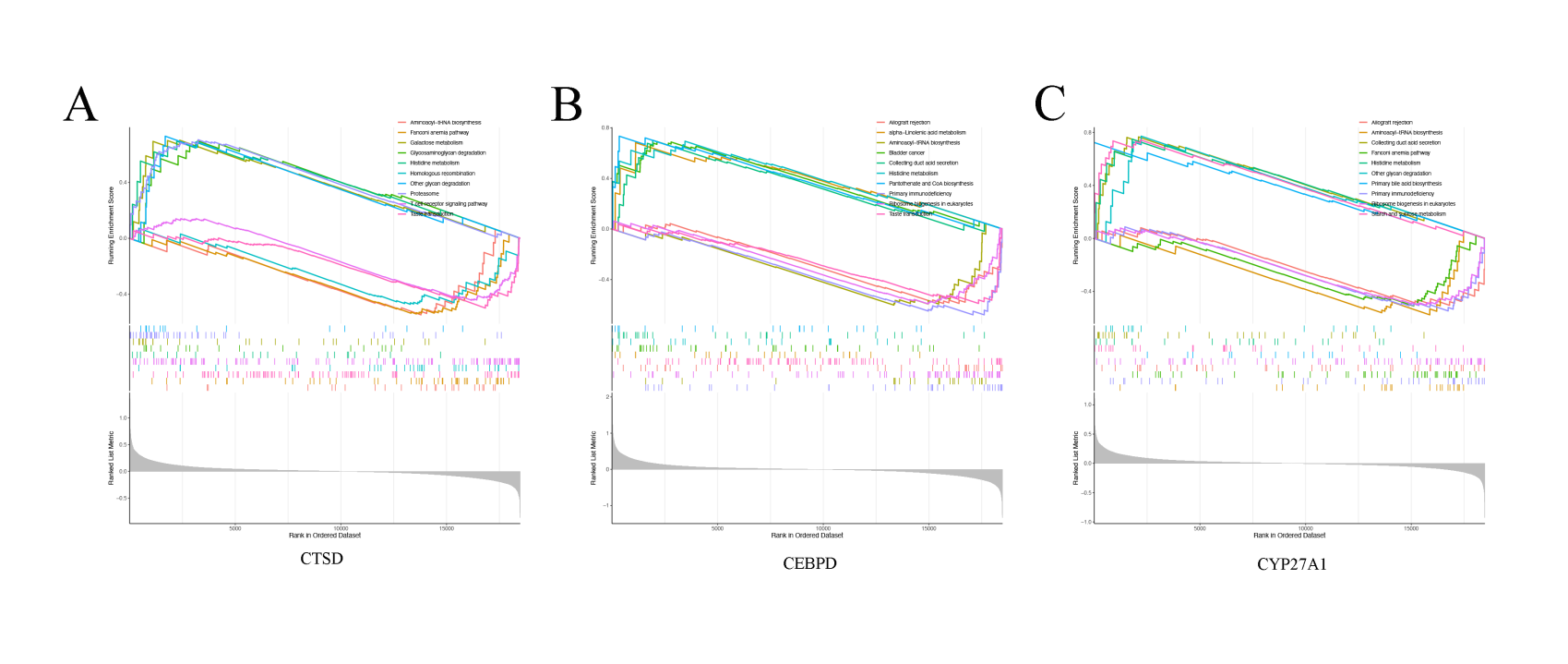


**Supplementary Figure 4.** Single-gene Gene Set Enrichment Analysis (GSEA) of test dataset (GSE62646+GSE123342). **(A)** single-gene GSEA of CTSD; **(B)** single-gene GSEA of CEBPD; **(C)** single-gene GSEA of CYP27A1. The lines in each figure indicate the top 10 enriched items:

Table

| **Supplementary Table 1.** Top 20 node genes ranked by Degree algorithm | | |
| --- | --- | --- |
| No. | Gene_name | Rank_score |
| 1 | STAT1 | 22 |
| 2 | IL6R | 8 |
| 3 | ANKRD22 | 6 |
| 4 | CYP27A1 | 6 |
| 5 | IL1RN | 6 |
| 6 | TIMP1 | 6 |
| 7 | IMPA2 | 4 |
| 8 | VDR | 4 |
| 9 | S100A11 | 4 |
| 10 | BCL3 | 4 |
| 11 | IRAK3 | 4 |
| 12 | BIRC3 | 4 |
| 13 | CTSD | 4 |
| 14 | GBP4 | 4 |
| 15 | CEBPD | 4 |
| 16 | GBP5 | 4 |
| 17 | JUNB | 4 |
| 18 | IFNGR1 | 4 |
| 19 | RGS2 | 4 |
| 20 | PLSCR1 | 2 |

| **Supplementary Table 2.** The prediction of target drugs for the management of IBD patients with the risk of CAD progression | | | | | | |
| --- | --- | --- | --- | --- | --- | --- |
| **Index** | **Name** | **P-value** | **Adjusted**  **P-value** | **Odds Ratio** | **Combined Score** | **Genes** |
| 1 | resveratrol CTD 00002483 | 5.12E-04 | 0.041274203 | 55197 | 418231.1847 | CTSD;CYP27A1;CEBPD |
| 2 | progesterone CTD 00006624 | 8.77E-04 | 0.041274203 | 54255 | 381927.2499 | CTSD;CYP27A1;CEBPD |
| 3 | pepstatin CTD 00001504 | 0.001798976 | 0.041274203 | 908.4545455 | 5741.921288 | CTSD |
| 4 | Cerebrosterol CTD 00001995 | 0.001798976 | 0.041274203 | 908.4545455 | 5741.921288 | CYP27A1 |
| 5 | Tamibarotene CTD 00002527 | 0.002296049 | 0.041274203 | 69.80251346 | 424.1595436 | CTSD;CEBPD |
| 6 | amodiaquine CTD 00005388 | 0.002398159 | 0.041274203 | 666.0666667 | 4018.416231 | CTSD |
| 7 | 4-Hydroxytamoxifen CTD 00000850 | 0.002521102 | 0.041274203 | 66.48287671 | 397.7709931 | CTSD;CEBPD |
| 8 | Brucinum CTD 00003015 | 0.002547917 | 0.041274203 | 624.40625 | 3729.253306 | CTSD |
| 9 | cytarabine CTD 00005743 | 0.002589763 | 0.041274203 | 65.55743243 | 390.4724634 | CYP27A1;CEBPD |
| 10 | 2-arachidonoylglycerol CTD 00003218 | 0.00269766 | 0.041274203 | 587.6470588 | 3476.150033 | CYP27A1 |
